# Transcriptomic Analysis of the Strain *Acidiplasma* sp. YE-1 During the Oxidation of Sulfide Minerals Pyrite and Arsenopyrite

**DOI:** 10.3390/ijms26199287

**Published:** 2025-09-23

**Authors:** Aleksandr Bulaev, Vitaly Kadnikov, Yulia Elkina, Aleksey Beletsky, Alena Artykova, Aleksandr Kolosoff, Nikolai Ravin, Andrey Mardanov

**Affiliations:** Research Center of Biotechnology, Russian Academy of Sciences, 119071 Moscow, Russia; vkadnikov@bk.ru (V.K.); yollkina@mail.ru (Y.E.); mortu@yandex.ru (A.B.); alena.artykov@gmail.com (A.A.); alexan-der_thechemist_kolosoff@mail.ru (A.K.); nravin@mail.ru (N.R.)

**Keywords:** differential gene expression, transcriptome, acidophilic archaea, *Acidiplasma*, biohydrometallurgy, pyrite, arsenopyrite, sulfocyanin

## Abstract

Extremely acidophilic iron- and sulfur-oxidizing bacteria and archaea are used in the processing of different sulfide ores and concentrates (biohydrometallurgical technologies); therefore, studying their metabolic pathways and regulation is an urgent task. Thus, the goal of this work was to compare differential gene expression in the thermoacidophilic archaeal strain, representative of the genus *Acidiplasma*, a predominant microbial group in bioleach reactors, during growth in the presence of ferrous iron and elemental sulfur as well as pyrite and arsenopyrite, which are the most widespread sulfide minerals, and to obtain novel data on the mechanisms of interaction of microorganisms and sulfide minerals. Transcriptomic analysis revealed metabolic pathways involved in ferrous iron and sulfur oxidation (key processes in sulfide mineral oxidation) and determined their expression dependence on different substrates. It was shown that the blue copper protein sulfocyanin may play an important role in both iron and sulfur oxidation, while sulfur oxidation also involves genes encoding well-known proteins for reduced inorganic sulfur compounds (RISC), sulfur oxygenase reductase (SOR), and thiosulfate quinone oxidoreductase (TQO). The results obtained in the present study may be used in further work to improve biohydrometallurgical technologies.

## 1. Introduction

Extremely acidophilic iron- and sulfur-oxidizing bacteria and archaea are used for processing different sulfide ores and concentrates (biohydrometallurgical technologies). Biohydrometallurgical processing is possible due to the microbially mediated destruction of sulfide minerals and may be implemented as in situ and heap leaching processes, as well as in stirred tank reactors [1,2,3]. Sulfide mineral oxidation is always performed by mixed microbial populations, which include several species of iron- and sulfur-oxidizing microorganisms [1,3]. Their composition may be affected by the composition of the oxidized ore and concentrate, temperature, pH, oxygen, and carbon availability, etc. [3]. Due to the self-heating of industrial-scale reactors and heaps, thermotolerant microorganisms, moderate thermophiles, and thermophiles usually predominate in these populations. These microorganisms include representatives of both bacteria and archaea: the bacteria *Leptospirillum* and *Sulfobacillus*, moderately thermophilic representatives of the genus *Acidithiobacillus* (*A*. *caldus*), and archaea of the family *Ferroplasmaceae* (genera *Acidiplasma* and *Ferroplasma*) [4,5,6,7,8,9,10,11,12,13,14,15,16,17,18,19,20].

Extremely acidophilic archaea of the genus *Acidiplasma* are moderately thermophilic heterotrophic microorganisms capable of oxidizing both iron and sulfur, which may predominate in microbial populations of laboratory- and industrial-scale bioleaching reactors and heaps, as well as in natural environments characterized by low pH values and high temperatures [15,21,22,23,24,25,26,27].

Representatives of the genus have an optimal temperature range of 45–55 °C and an optimal pH range of 1.0–1.6 [15,21,22,23].

Genomes of known representatives of the genus *Acidiplasma* have been sequenced [24,25,26,27]. These genomes are 1.7–1.8 Mbp long with a GC content of around 34%. Genome analysis revealed the presence of the gene encoding sulfocyanin, a blue copper-containing protein that plays a key role in iron oxidation, as well as genes for sulfur oxidoreductase and sulfate adenyltransferase (enzymes involved in sulfur oxidation), genes involved in the transport and catabolism of organic compounds, and genes of the 3-hydroxypropionate/4-hydroxybutyrate cycle [24]. Despite genome sequencing, there are no data on gene expression in *Acidiplasma* spp.

Evolutionary patterns of the genus *Acidiplasma* and other archaea of the order *Thermoplasmatales* were considered in [27]. It was proposed that *Ferroplasma*, *Acidiplasma*, and *Picrophilus* acquired about 500 genes, which were absent in the common ancestors of *Thermoplasmatales*, and lost 170 genes present in the ancestors. Among these, both the gained and lost genes included components of metal ion transport systems, which are important for acidophiles, considering the specific environments they inhabit.

Most gene expression studies under different conditions of acidophilic microorganisms used in biohydrometallurgy have been performed on the “model” microorganism *Acidithiobacillus ferrooxidans.* In this bacterium, metabolic pathways of iron and sulfur oxidation, mechanisms of resistance to heavy metals, and regulation of intracellular pH have been studied [28,29,30,31,32].

It should be noted that methods enabling the study of gene expression are used to determine the mechanisms of interaction between acidophilic microorganisms and sulfide minerals. For example, in proteomic and transcriptomic studies [33], it was shown that gene expression profiles of *A*. *ferrooxidans* cells contacted with minerals of similar chemical composition, chalcopyrite (CuFeS_2_) and bornite (Cu_5_FeS_4_), differed. This suggests that despite the similarity in chemical composition of these minerals, the metabolic pathways involved in their oxidation differ. Another important conclusion from these studies is that, in addition to oxidation system proteins, proteins involved in transport systems and the synthesis of some organic monomers also play an important role in mineral oxidation (the authors could not explain this phenomenon).

Several studies have shown that oxidative stress-response systems also play an important role in the biooxidation of sulfide minerals. For instance, [34] demonstrated that during pyrite biooxidation by *A. ferrooxidans*, expression increased for genes involved in the oxidative stress response.

In [35], proteome analysis was used to analyze the mechanisms employed by the *Sulfobacillus thermotolerans* bacterium to protect itself from the toxic effects of gold-bearing pyrite-arsenopyrite ore concentrate. It was shown that oxidative stress protection systems play an important role in the bacterium’s resistance to the stress conditions induced by the concentrate.

Ref. [36] studied the adaptation of *L. ferriphiluum* to chalcopyrite oxidation using transcriptomics and proteomics. It was shown that during chalcopyrite oxidation, expression increased for genes conferring copper resistance, as well as those responsible for chemotaxis and cell motility.

Thus, the interaction of microorganisms with sulfide minerals is actively studied, including using “OMIC” methods, but is still an unexplored process in many aspects. Current studies do not yet enable systemic analysis of definitive conclusions regarding the specific metabolic pathways involved in the oxidation of different sulfide minerals by different microorganisms.

In the present work, we studied gene expression in the strain *Acidiplasma* sp. YE-1, one of the predominant microorganisms in the microbial population of a laboratory-scale biooxidation reactor during processing pyrite-arsenopyrite concentrate [37], during the growth in a nutrient medium containing different substrates as electron donors: ferrous iron, elemental sulfur, pyrite, and arsenopyrite. According to generally accepted models of sulfide mineral biooxidation [38], during bioleaching, microorganisms oxidize ferrous iron and sulfur compounds to produce the strong oxidizer, ferric ions, as well as sulfuric acid, which then interact with sulfide minerals, leading to leaching. This mechanism is called the “indirect mechanism” and is considered the primary mechanism of sulfide mineral biooxidation [39,40]. At the same time, different sulfide minerals may be oxidized by different specific mechanisms. For example, pyrite is oxidized via so-called thiosulfate mechanisms, whereas most other sulfide minerals, including arsenopyrite, follow the polysulfide pathway [38,39,40,41,42]. These mechanisms involve the formation of different key intermediates. During biooxidation of pyrite, the mineral is primarily oxidized by ferric ions, resulting in thiosulfate formation from the sulfur moiety; thiosulfate is then also oxidized by ferric ions. In the polysulfide mechanism, polysulfides and elemental sulfur are formed, which require oxidation by sulfur-oxidizing microorganisms. Thus, different enzymatic pathways may be involved in the oxidation of different minerals. Furthermore, different cellular mechanisms may be engaged in the interaction between microbial cells and minerals, including those promoting extracellular polymeric substance (EPS) formation and oxidative stress responses [34,42]. However, comparative data on gene expression in acidophilic microorganisms during growth on various sulfide minerals are limited. Therefore, the precise mechanisms of microbial–mineral interactions are not completely understood.

Thus, the goal of this work was to compare gene expression in the polyextremophilic strain *Acidiplasma* sp. YE-1 during the growth in the presence of ferrous iron (control) and elemental sulfur, as well as pyrite and arsenopyrite (the most widespread sulfide minerals), and to obtain novel data on the mechanisms of interaction of microorganisms and sulfide minerals. Ferrous iron oxidation is well-known for being a representative of *Acidiplasma* [21,22,23,24]. Sulfur oxidation as a representative of the genus *Acidiplasma* was shown in our previous work [43,44]. In the present work, we compared gene expression during growth, both in the presence of ferrous iron (control) and elemental sulfur, to reveal the genes involved in the oxidation of these electron donors.

## 2. Results

### 2.1. Growth of Acidiplasma sp. YE-1 with Different Electron Donors

**Figure 1:**
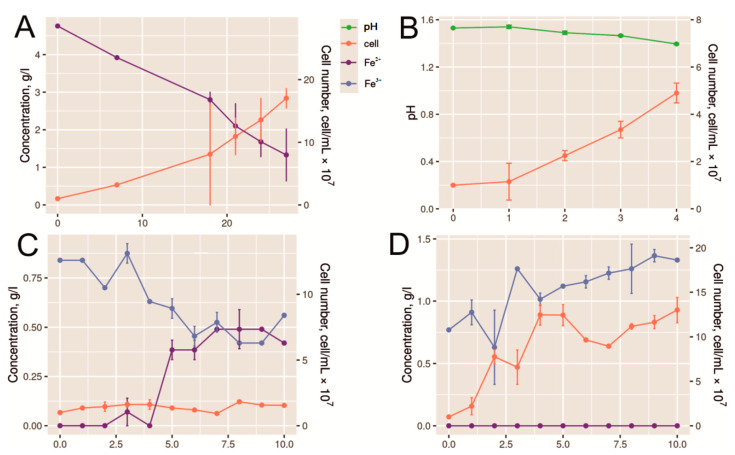
Growth of *Acidiplasma* sp. YE-1 in different conditions. (**A**) Change in ferrous iron ion concentrations and cell number during the growth in the medium containing ferrous sulfate as electron donor; (**B**) Change in pH value and cell number during the growth in the medium containing sulfur (S^0^) as electron donor; (**C**) Change in iron ion concentrations (Fe(III) and Fe(II) and cell number during pyrite bioleaching; (**D**) Change in iron ion concentrations (Fe(III) and Fe(II) and cell number during arsenopyrite bioleaching. The *x*-axis indicates sampling hours (**A**) and days (**B**–**D**).

Growth curves of the strain in media containing ferrous iron (Fe^2+^) and sulfur (S^0^) as electron donors are shown in Figure 1A,B. Growth in the ferrous iron-containing medium resulted in the highest cell number. Ferrous iron was oxidized for 28 h, and the cell number reached 1.7 × 10^8^ cell/mL. Growth in the medium with sulfur was accompanied by a pH decrease due to sulfate formation. The cell number increased up to 5.0 × 10^7^ cell/mL.

The conducted analysis of microbial growth in medium containing pyrite and arsenopyrite showed that the maximum cell number was reached on the fourth day of growth (Figure 1C,D). At that point, the process of active growth ceased, indicating the end of the exponential growth phase. Thus, for transcriptome analysis, cells were harvested after 4 days of growth on pyrite and arsenopyrite.

It should be noted that the growth in the presence of pyrite was accompanied by an accumulation of ferrous iron. This suggests that the oxidizing activity of the strain was low. This observation is consistent with that from our previous work, which demonstrated a low rate of pyrite oxidation by a pure culture of the strain *Acidiplasma* sp. MBA-1 [45].

In contrast, during growth in the presence of arsenopyrite, the ferrous iron concentration was below the detection limit, while ferric iron content increased throughout arsenopyrite leaching (Figure 1D). Thus, ferrous iron biooxidation was not inhibited by arsenopyrite.

### 2.2. General Characteristics of Transcriptomic Data

We compared changes in the transcription profiles of *Acidiplasma* sp. YE-1 genes during growth of pure cultures with different electron donors (Fe^2+^, S^0^) or on pyrite and arsenopyrite. Growth with ferrous iron (Fe^2+^) as the electron donor served as the control. A total of 2.6 billion reads were obtained across the 12 samples, with a minimum of 116 million reads per sample. Ribosomal RNAs comprised 47–74% of the total reads. Consequently, after removing low-quality reads and ribosomal RNA reads, the reads mapped to protein-coding sequences from *Acidiplasma* sp. YE-1 accounted for at least 31 million reads per sample (Table S1). Expression of all 1743 annotated protein-coding genes on the chromosome was detected under every growth condition (Table S2).

**Figure 2:**
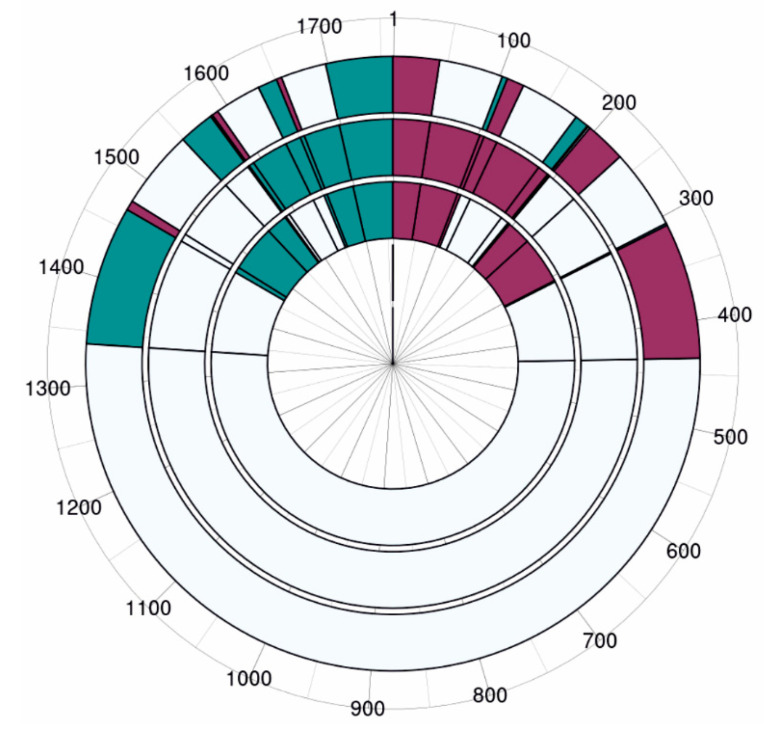
Circular diagram of DEGs identified under sulfur, pyrite, and arsenopyrite growth conditions versus the control (ferrous iron-grown *Acidiplasma* sp. YE-1). The outer ring represents DEGs during sulfur growth, the middle ring DEGs during pyrite growth, and the inner ring DEGs during arsenopyrite growth. Cherry-colored sectors: DEGs with log2FoldChange ≥ 1 and p-adj ≤ 0.05 (upregulated); turquoise sectors: DEGs with log2FoldChange ≤ −1 and p-adj ≤ 0.05 (downregulated).

Differentially expressed genes (DEGs) were identified for each growth condition relative to the control. The largest number of DEGs (510 genes; 248 upregulated and 262 downregulated), defined as showing a >2-fold change with statistical significance (p-adj < 0.05), occurred during growth on elemental sulfur (S^0^). For growth on pyrite and arsenopyrite, 376 DEGs (196 upregulated, 180 downregulated) and 454 DEGs (224 upregulated, 230 downregulated) were observed, respectively (Figure 2). The higher number of DEGs during sulfur growth compared to growth on minerals suggests that sulfur utilization involves unique regulatory pathways, whereas iron oxidation mechanisms appear conserved during growth on pyrite and arsenopyrite.

During cultivation with sulfur or on sulfur-containing minerals, five genes were identified exhibiting transcription levels that increased more than eight-fold under all three conditions relative to the control: genes encoding hypothetical proteins (WMT55490, WMT54948), sulfur oxygenase/reductase (WMT54692), aminodeoxychorismate/anthranilate synthase (WMT54501), and a metal-dependent transcriptional regulator (WMT54903). Additionally, 5 genes showed transcription levels that decreased more than 14-fold relative to the control under all conditions: an MFS transporter (WMT55463), peroxiredoxins (WMT55172, WMT55173), formylmethanofuran dehydrogenase subunit E (WMT55891), and asparaginyl-tRNA synthetase (WMT55614).

### 2.3. KEGG and COG Annotation and Classification of DEGs

Using KEGG and COG databases, we performed an analysis of differentially expressed genes (DEGs) during growth of *Acidiplasma* YE-1 in the presence of sulfur, pyrite, and arsenopyrite, compared to control conditions (growth on ferrous iron).

**Figure 3:**
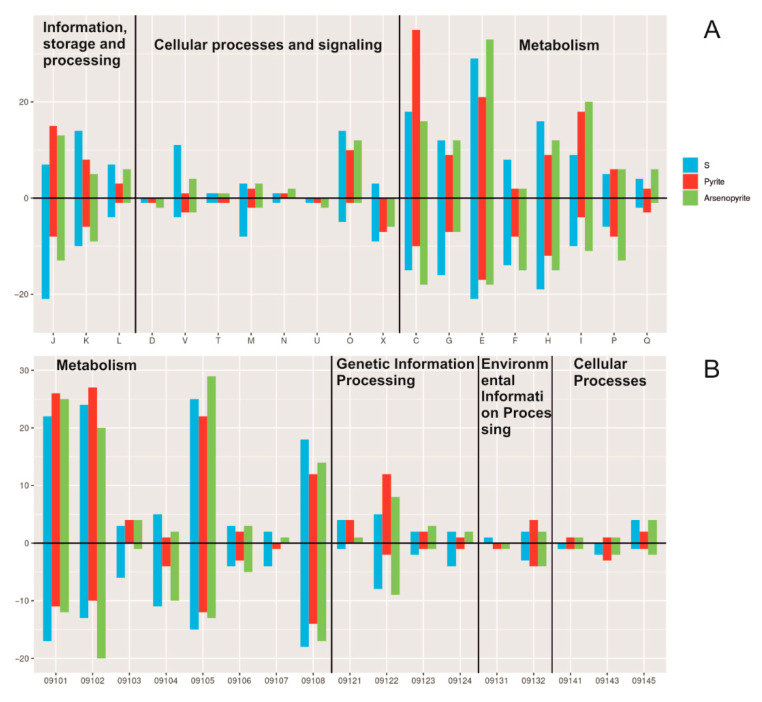
COG (**A**) and KEGG (**B**) distribution of differentially expressed genes (DEGs) in the transcriptomes *Acidiplasma* sp. YE-1. COG categories: J—Translation, ribosomal structure and biogenesis; K—Transcription; L—Replication, recombination and repair; D—Cell cycle control, cell division, chromosome partitioning; V—Defense mechanisms; T—Signal transduction mechanisms; M—Cell wall/membrane/envelope biogenesis; N—Cell motility; U—Intracellular trafficking, secretion, and vesicular transport; O—Posttranslational modification, protein turnover, chaperones; X—Mobilome: prophages, transposons; C—Energy production and conversion; G—Carbohydrate transport and metabolism; E—Amino acid transport and metabolism; F—Nucleotide transport and metabolism; H—Coenzyme transport and metabolism; I—Lipid transport and metabolism; P—Inorganic ion transport and metabolism; Q—Secondary metabolites biosynthesis, transport, and catabolism. KEGG pathway modules: 9101—Carbohydrate metabolism; 9102—Energy metabolism; 9103—Lipid metabolism; 9104—Nucleotide metabolism; 9105—Amino acid metabolism; 9106—Metabolism of other amino acids; 9107—Glycan biosynthesis and metabolism; 9108—Metabolism of cofactors and vitamins; 9121—Transcription; 9122—Translation; 9123—Folding, sorting, and degradation; 9124—Replication and repair; 9131—Membrane transport; 9132—Signal transduction; 9141—Transport and catabolism; 9143—Cell growth and death; 9145—Cellular community—prokaryotes.

Most of the COG category was related to metabolic functions, such as energy production and conversion, amino acid transport and metabolism, and coenzyme transport and metabolism (Figure 3).

COG database analysis assigned the majority of DEGs to metabolic categories, with the categories “Energy production and conversion” and “Amino acid transport and metabolism” being predominant. Notably, within the “Energy production and conversion” category, the number of upregulated DEGs during pyrite cultivation was twice that observed on sulfur or arsenopyrite. Given that the pure *Acidiplasma* YE-1 culture exhibits reduced growth on pyrite, this transcriptional increase potentially reflects microbial adaptation to this less favorable substrate.

KEGG pathway analysis revealed that the largest proportion of DEGs across all substrates (arsenopyrite, pyrite, and elemental sulfur) were assigned to the “Metabolism” and “Genetic Information Processing” categories (Figure 3). Within these, the predominant functional groups were “Carbohydrate metabolism” and “Energy metabolism”, suggesting enhanced glycolytic activity, respiration, and energy production. Additionally, DEGs implicated in amino acid, lipid, cofactor, and vitamin metabolism were highly represented, consistent with upregulated biosynthetic processes and cellular adaptation to mineral-induced stress.

Genes implicated in genetic information processing—including transcription, translation, protein folding, and DNA repair—exhibited consistently high expression levels across all tested substrates (Figure 3). Furthermore, the elevated expression of genes associated with membrane transport and signal transduction, particularly during growth on arsenopyrite, suggests the activation of cellular adaptation and stress-response mechanisms.

A comprehensive analysis of KEGG enrichment results for *Acidiplasma* sp. transcriptomes under different mineral conditions (pyrite, arsenopyrite, and sulfur) revealed distinct functional gene expression patterns associated with each substrate (Table S3).

Under pyrite conditions, upregulated DEGs were significantly enriched in central metabolic pathways, including the citrate cycle (TCA cycle; ko00020), carbon fixation (ko00720), and sulfur metabolism (ko00920), indicating enhanced energy generation and autotrophic CO_2_ assimilation. Enrichment of sulfur metabolism genes implies increased activity of sulfur transformation enzymes, consistent with *Acidiplasma*’s adaptation to sulfur-rich environments. Conversely, downregulated DEGs associated with DNA replication and repair, transport, and signaling pathways suggest a broad suppression of cellular activity under pyrite (Table S3). This pattern may reflect a transition to a lower metabolic state, potentially due to energetic or nutritional constraints imposed by pyrite as a growth substrate.

Growth on arsenopyrite resulted in significantly reduced representation of DEGs in the “Transporters” pathway (ko02000) and functionally linked signaling/cellular process genes (ko09193). This transcriptional suppression likely indicates systemic metabolic repression, potentially resulting from arsenic toxicity or an adaptive energy conservation strategy during arsenic stress.

Growth on sulfur resulted in significant enrichment of DEGs implicated in the bacterial defense system pathway (ko02048). This upregulation suggests activation of protective mechanisms, including CRISPR-Cas systems (Table S3).

### 2.4. Metabolic Changes in Acidiplasma YE-1 Across Growth Conditions

#### 2.4.1. Metabolism of Carbon and Carbohydrates

Analysis of carbon metabolism genes (glycolysis, tricarboxylic acid cycle, pentose phosphate pathway) in *Acidiplasma* YE-1 cultivated on different substrates (sulfur, pyrite, arsenopyrite) demonstrated no significant changes in the expression profiles of these pathways across growth conditions relative to the control (ferrous iron). Within glycolysis-related expression changes, two notable alterations were observed: significant upregulation of fructose-1,6-bisphosphate aldolase (*fbp*) during growth on sulfur and arsenopyrite, and downregulation of triosephosphate isomerase (tpi), specifically on sulfur. Transcription of the key pentose phosphate pathway gene ribose 5-phosphate isomerase A (*rpiA*) exhibited a significant decrease across all substrate growth conditions relative to the control (Table S4). Some changes at the level of individual genes have been detected in the tricarboxylic acid cycle (TCA). In particular, the expression level of the 2-oxoglutarate/2-oxoacid ferredoxin oxidoreductase (*korAB*) gene was increased when cultured on pyrite, arsenopyrite, and sulfur compared to ferrous iron. At the same time, expression levels of the succinate dehydrogenase/fumarate reductase flavoprotein subunit genes (*sdhA*, *frdA*) and the fumarate reductase iron-sulfur subunit gene (*frdB*) decreased significantly. Moreover, transcriptional reduction relative to the control exceeded two-fold in mineral-grown cultures (pyrite and arsenopyrite) compared to sulfur-grown cultures (Table S4).

Biological fixation of inorganic carbon, one of the oldest and most significant in terms of the volume of biosynthetic processes in nature, is carried out through at least seven metabolic pathways [46,47,48]. The ability of microorganisms to grow using atmospheric CO_2_ is key for biomining processes, as it avoids the need to add organic compounds during bioleaching and biooxidation. It has been proposed that inorganic carbon fixation in *Acidiplasma* YE 1 occurs via the 3-Hydroxypropionate/4-Hydroxybutyrate (3HP-4HB) cycle [48]. Our genomic analysis identified several genes associated with this pathway, including those encoding methylmalonyl-CoA mutase, 4-hydroxybutyrate-CoA ligase, 4-hydroxybutyryl-CoA dehydratase, enoyl-CoA hydratase, and acetyl-CoA C-acetyltransferase. The transcription of these genes showed only minor variations under different growth conditions. However, a number of key enzymes essential for the 3HP-4HB cycle were not identified in the genome. These include acetyl-CoA/propionyl-CoA carboxylase, malonyl-CoA/succinyl-CoA reductase, 3-hydroxypropionate dehydrogenase, 3-hydroxypropionyl-CoA synthetase, methylmalonyl-CoA epimerase, succinyl-CoA reductase, and succinate semialdehyde reductase. The absence of these essential genes suggests that the 3HP-4HB cycle is incomplete and likely non-functional in this microorganism.

#### 2.4.2. Sulfur and Iron Metabolism

Transcriptomic analysis of the archaeon *Acidiplasma* YE-1 grown on elemental sulfur, pyrite, or arsenopyrite revealed an upregulation of genes associated with sulfur metabolism compared to growth on ferrous iron. These included genes encoding sulfur oxygenase/reductase, sulfide oxidoreductase, and thiosulfate oxidoreductase. Among these, sulfur oxygenase/reductase (SOR) is a key enzyme in the archaeal thermoacidophilic sulfur oxidation pathway [49]. SOR catalyzes the simultaneous oxidation and reduction of sulfur (disproportionation) via an oxygenase reaction under aerobic, high-temperature conditions. *Acidiplasma* YE-1 also encodes enzymes that oxidize the products of the SOR reaction (sulfite, thiosulfate, and sulfide), thereby coupling sulfur oxidation to the electron transport chain and substrate phosphorylation [50]. Furthermore, a gene cluster encoding the two subunits of thiosulfate:quinone oxidoreductase (TQO) was identified in its genome. The expression of these genes was upregulated during growth on elemental sulfur (more than 20-fold), pyrite (2.3-fold), or arsenopyrite (1.94-fold) compared to ferrous iron (Table S4).

In bacteria, iron oxidation pathways involve key components such as c-type cytochromes, copper-containing proteins, iron-sulfur clusters, and terminal oxidases [51]. In contrast, the mechanisms of electron transfer from iron to oxygen in archaea remain poorly characterized. In members of the *Sulfolobales* order (e.g., *Metallosphaera* and *Sulfolobus*), this process is proposed to be facilitated by membrane-bound redox proteins encoded by the fox operon [52]. Additionally, Sox and Dox complexes implicated in iron oxidation have been identified in *Metallosphaera* species [53]. In contrast, such complexes are absent in representatives of the *Ferroplasma*, *Picrophilus*, and *Acidiplasma* genera [27].

**Figure 4:**
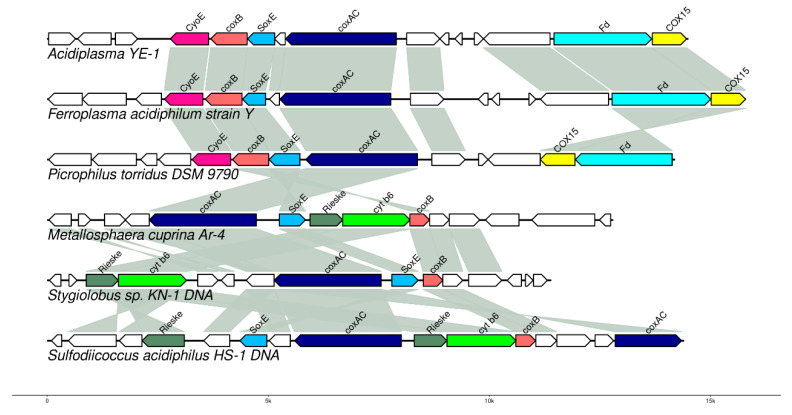
Organization of the sulfocyanine-related gene cluster. COX15—cytochrome c oxidase assembly factor, *coxAC*—cytochrome c oxidase polypeptide I/III, *coxB*—cytochrome c oxidase polypeptide II, *CyoE*—protoheme IX farnesyltransferase, *cyt b6*—cytochrome b6, *Fd*—ferredoxin, *Rieske*—Rieske (2Fe-2S) protein, *SoxE*—sulfocyanin, white-colored box—hypothetical protein.

Genome analysis of *Acidiplasma* YE-1 identified a gene cluster (locus WMT54265–WMT54276) putatively involved in iron oxidation (Figure 4; Table S2). This cluster encodes a blue copper protein, sulfocyanin (WMT54267), which may function in concert with cytochromes to facilitate iron oxidation. Notably, high expression levels of a sulfocyanin homolog were previously reported in the bacterium *Sulfobacillus thermosulfidooxidans* ST during growth on ferrous iron [54]. Intriguingly, in *Acidiplasma* YE-1, the expression of this gene cluster was higher during growth on elemental sulfur than on ferrous iron, decreased on pyrite, and remained unchanged on arsenopyrite (Table S2). The precise physiological role of sulfocyanin remains to be fully elucidated. Although its involvement in archaeal iron metabolism has long been proposed [51,55], direct experimental evidence exists only for *Ferroplasma acidiphilum* [56]. In *Acidiplasma* YE-1, the gene cluster encoding sulfocyanin is similar to that found in *Ferroplasma acidiphilum* strain Y. A cluster of similar architecture is also present in the genome of *Picrophilus torridus*, albeit with two key rearrangements: (1) the absence of an open reading frame (ORF) between the *soxE* and *coxAC* genes, and (2) an inversion, placing the ferredoxin (*fd*) and *COX15* genes in reverse order (Figure 4). Notably, *Picrophilus torridus* does not oxidize ferrous iron, despite possessing a sulfocyanin homolog. In *P. torridus*, the genes encoding this putative respiratory complex are located on a genomic island. This suggests they may have been acquired via horizontal gene transfer, similar to the rusticyanin/sulfocyanin genes identified in *C. divulgatum* S5 and *F. acidiphilum* [57].

#### 2.4.3. Oxidative Phosphorylation

Analysis of oxidative phosphorylation genes revealed that the transcriptional levels of most genes during growth on elemental sulfur, pyrite, or arsenopyrite were comparable to those observed during cultivation on ferrous iron. However, the expression of subunits A, B, C, D, and I of the NADH oxidoreductase complex was upregulated more than two-fold during growth on pyrite. The transcription of complex II (succinate dehydrogenase) was downregulated on pyrite and arsenopyrite but remained unchanged on sulfur. For complex IV (cytochrome c oxidase), expression decreased more than two-fold on arsenopyrite and increased on sulfur, while no significant change was observed on pyrite compared to ferrous iron (Table S4).

#### 2.4.4. Oxidative Stress

The oxidation of ferrous iron, sulfur, or reduced sulfur compounds in aerobic organisms is coupled to the respiratory chain, where oxygen serves as the terminal electron acceptor, driving proton translocation across the membrane. Among the upregulated genes were those encoding thioredoxin and rubrerythrin, both of which are key enzymes involved in cellular reduction processes and protection against oxidative stress. Thioredoxin facilitates the reduction of disulfide bonds and helps maintain redox homeostasis, whereas rubrerythrin acts as an antioxidant, detoxifying free radicals and peroxides. The increased expression of these proteins in *Acidiplasma* YE-1 indicates the activation of defense mechanisms against stress induced by toxic metal ions and other adverse factors. This suggests that bacterial adaptation to pyrite and arsenopyrite involves the mobilization of robust antioxidant systems to mitigate their toxic effects.

## 3. Discussion

In the present study, the dependence of the expression of the genes involved in different metabolic pathways in polyextremophilic archaea *Acidiplasma* YE-1 on the presence of various oxidized substrates was studied. The main findings of the study are as follows:

- Metabolic pathways involved in ferrous iron and sulfur oxidation, which play a key role in sulfide mineral oxidation, were identified, and the dependence of their expression on the presence of different substrates was determined.
- In *Acidiplasma* sp. YE-2, sulfocyanin may play an important role both in iron and sulfur oxidation, while sulfur oxidation also involves genes well-known proteins participating in the oxidation of reduced inorganic sulfur compounds (RISC), SOR, and TQO.
- The presence of pyrite led to a decrease in sulfocyanin expression level, which was accompanied by iron oxidation inhibition that, in turn, resulted in the low rate of pyrite bioleaching.

Thus, this study provides novel data on the regulation of metabolic pathways in polyextremophilic archaea involved in bioleaching. A comparison with previous studies on acidophilic microorganisms suggests that the metabolic pathways and their regulation in representatives of the genus *Acidiplasma* exhibit unique features.

The differential gene expression of various acidophilic microorganisms involved in sulfide mineral bioleaching has been studied with respect to the oxidized substrate provided. Numerous studies have focused on bacteria of the genus *Acidithiobacillus*, the most extensively characterized acidophilic iron- and sulfur-oxidizers. Several investigations have specifically examined representatives capable of oxidizing both ferrous iron (Fe^2+^) and reduced inorganic sulfur compounds (RISCs). For instance, clear differences in gene expression were demonstrated in *Acidithiobacillus ferrooxidans* [32,58,59] and *A. ferriphilus* [60] during growth on ferrous iron versus sulfur. Growth on ferrous iron led to the upregulation of key genes involved in Fe^2+^ oxidation, including those encoding rusticyanin and the petI gene cluster in *A. ferrooxidans* [32,58] and *A. ferriphilus* [60], respectively. In contrast, growth in the presence of RISCs resulted in the upregulation of genes encoding enzymes for RISC oxidation [32,58,59,60]. For bacteria of the genus *Acidithiobacillus*, differential gene expression in response to the presence of sulfide minerals was also studied [33,34,60,61,62].

The authors of [34] demonstrated a change in gene expression in *A. ferrooxidans* during growth on pyrite. The proteomic analysis revealed higher levels of proteins associated with the oxidative stress response and ROS detoxification. The study by [33] revealed different gene expression profiles in *A. ferrooxidans* after 24 h exposure to chalcopyrite (CuFeS_2_) or bornite (Cu_5_FeS_4_). It was shown that the expression of genes encoding both transport system proteins and proteins involved in the synthesis of certain organic monomers was upregulated.

In the sulfur-oxidizing bacterium *A*. *thiooxidans*, genes involved in RISC oxidation were significantly upregulated during exposure to chalcopyrite [61]. The authors proposed that this bacterium may adapt to the stress conditions by enhancing sulfur oxidation to generate more energy.

In the presence of arsenopyrite, representatives of the genus *Acidithiobacillus* increased the expression of *ars* operon genes (which are involved in arsenic resistance), as well as genes encoding transmembrane ion transport proteins, stress-response factors, enzymes for inorganic polyphosphate accumulation and urea catabolism, tryptophan biosynthesis machinery, and proteins responsible for ferrous iron and RISC oxidation [62]. In other acidophilic bacteria, differential gene expression in response to sulfide minerals has also been observed.

In [36], the adaptations of *Leptospirillum ferriphilum* to growth on chalcopyrite were studied. In comparison to growth on ferrous iron, cultivation in the presence of chalcopyrite triggered the upregulation of several gene groups, including those involved in ferrous iron oxidation, carbon dioxide fixation, and heavy metal resistance (e.g., copper efflux systems) [36].

The mechanisms protecting the bacterium *S. thermotolerans* from the toxic effects of a gold-bearing pyrite-arsenopyrite ore concentrate were analyzed using proteomics. The analysis demonstrated that systems for oxidative stress protection play a key role in the bacterium’s resistance to the stress conditions induced by the concentrate [35].

The adaptive mechanisms of *S. thermotolerans* to the sulfide ore concentrate were studied in [35]. Proteins involved in sulfur metabolism were upregulated, including sulfide:quinone oxidoreductase, cysteine desulfurase, and S-adenosylmethionine synthetase. The synthesis of stress-response proteins was also enhanced.

Differential gene expression has also been investigated in acidophilic archaea, specifically representatives of the family *Ferroplasmaceae* and the order *Sulfolobales*. The protein complexes involved in ferrous iron and RISC oxidation in these archaea often differ from their bacterial counterparts [49,50,51,56,63]. However, similar proteins can occur in both domains due to horizontal gene transfer [64,65,66].

The iron oxidation respiratory chain has been studied in archaea of the genus *Ferroplasma*, which are closely related to *Acidiplasma*. Studies have demonstrated that the blue copper protein sulfocyanin is a key component of this chain, directly involved in ferrous iron (Fe^2+^) oxidation [51,56,64,65,67,68,69]. This protein is located on the exterior side of the cytoplasmic membrane, where it directly oxidizes Fe^2+^ and functions as part of a complex that includes an aa_3_-type cytochrome oxidase [51,63]. Proteomic analyses of *F. acidarmanus* confirmed sulfocyanin’s role in iron oxidation, showing its upregulation during growth on ferrous iron compared to media containing only organic substrates [67]. Under anaerobic conditions and during biofilm formation, other respiratory chain proteins involved in ferric iron (Fe^3+^) reduction coupled to organic compound oxidation may be upregulated [68,69].

Sulfocyanin has also been identified in archaea related to *Ferroplasma* and *Acidiplasma*, as well as in the heterotrophic species *Picrophilus* and *Cuniculiplasma*, which lack iron oxidation activity [57,64]. In representatives of *Ferroplasma*, *Picrophilus*, and *Cuniculiplasma*, the genes encoding the iron oxidation electron transport chain are located on genomic islands, suggesting acquisition via lateral gene transfer [57,64]. Furthermore, sulfocyanin exhibits sequence similarity to rusticyanin from *Acidithiobacillus* and to SoxE (a sulfocyanin homolog) from *Sulfolobus* [56].

Although sulfocyanin has been identified in thermophilic archaea of the order *Sulfolobales*, its role in ferrous iron oxidation in these organisms has not been clearly demonstrated [51,56]. Research indicates that in *Sulfolobales* archaea, proteins encoded by the *fox* gene cluster are likely the key components of ferrous iron oxidation complexes. This is supported by differential expression analyses showing that these genes are upregulated during growth on ferrous iron [51,52,56,70]. In *Sulfolobus acidocaldarius*, sulfocyanin is a component of a respiratory chain that includes the SoxABCD and SoxM supercomplexes, which are analogs of the mitochondrial cytochrome pathway [71,72]. It has been proposed that sulfocyanin may play a universal role in electron transfer [52].

Studies of sulfur oxidation pathways in archaea of the order *Sulfolobales* have revealed that sulfur oxygenase reductase (SOR) is a key enzyme in this process [49,50]. SOR catalyzes the disproportionation of elemental sulfur, producing thiosulfate, sulfite, and sulfide. Its expression is upregulated in the presence of sulfur [70]. Although SOR was initially characterized in archaea, it has also been identified in sulfur-oxidizing bacteria, such as those from the genus *Acidithiobacillus* [65].

**Figure 5:**
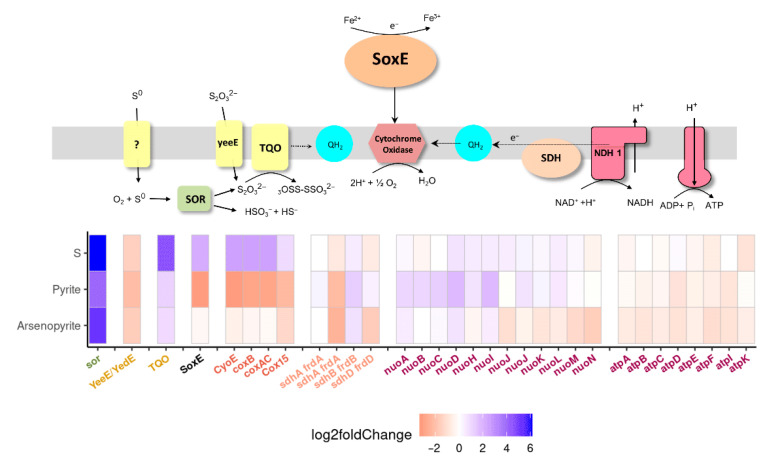
Putative pathways for ferrous iron and sulfur oxidation in *Acidiplasma* sp. YE-1. SoxE—sulfocyanin, SOR—sulfur oxygenase/reductase, TQO—thiosulfate:quinone oxidoreductase, QH_2_—quinol, NDH 1—NADH dehydrogenase, yeeE—thiosulfate uptake protein, SDH—complex II. Gene expression heatmap of key genes.

In the present work, genomic and transcriptomic analyses of an *Acidiplasma* representative revealed the involvement of known archaeal pathways in ferrous iron and sulfur oxidation. The upregulation of genes encoding sulfur oxygenase reductase (SOR) and thiosulfate:quinone oxidoreductase (TQO) was observed during growth on elemental sulfur, pyrite, and arsenopyrite compared to ferrous iron (Figure 5). This finding is consistent with the known functions of these enzymes in sulfur metabolism.

Notably, sulfocyanin—a key protein in the ferrous iron oxidation pathway of the related genus *Ferroplasma*—was also upregulated in *Acidiplasma* during growth on sulfur, suggesting potential additional functions beyond iron oxidation. In contrast, its expression decreased during growth on pyrite (Figure 5). This downregulation may contribute to the strain’s weak pyrite leaching activity, as ferrous iron oxidation is a critical step in this process [38,39].

The reason for the decreased sulfocyanin synthesis on pyrite remains unclear. Elucidating this mechanism requires further investigation due to its significant practical and scientific implications.

In the present study, we revealed that the genome of *Acidiplasma* YE-1 contains a subset of genes encoding enzymes of the 3-hydroxypropionate/4-hydroxybutyrate (3HP/4HB) cycle. These genes were expressed regardless of the growth substrate. The absence of the complete pathway is consistent with the fact that *Acidiplasma* representatives are generally considered heterotrophic [21,22,23,24]. The role of carbon dioxide fixation in both *Acidiplasma* and *Ferroplasma* has remained poorly understood, as these archaea require organic nutrients for growth. Weak carbon dioxide fixation has been demonstrated for some strains of *F. acidiphilum* [73]. Furthermore, a putative “chimeric pathway” for CO_2_ fixation has been proposed for *F. acidarmanus* fer1 and *Ferroplasma* types I and II. This pathway is thought to incorporate steps from both the reductive acetyl-CoA (Wood-Ljungdahl) pathway and the serine cycle [74].

Thus, this study provides novel data on the metabolic pathways of an *Acidiplasma* representative. As this genus plays an important role in the industrial-scale bioleaching of sulfide ores and concentrates, these findings could potentially be used to develop advanced biohydrometallurgical technologies based on synthetic biology and genetic engineering [75,76,77].

Currently, genetically engineered microorganisms involved in biomining are not used in practice. The application of genetic tools has been primarily studied in the model organism *A. ferrooxidans*, which is capable of both iron and sulfur oxidation; however, these studies have been confined to laboratory settings [75,76]. To date, there are no documented examples of genetically modified acidophilic strains being used in practical applications. Nevertheless, advances in synthetic biology and genetic engineering are expected to overcome key limitations in biohydrometallurgy by enabling the development of strains with enhanced properties, such as a faster growth rate and higher tolerance to heavy metals, acidity, and salinity.

For instance, the present work characterizes a representative of the genus *Acidiplasma*, which is known for its tolerance to high temperature, acidity, and ferric iron concentration [21,22,23,24,78]. We demonstrate a downregulation of key iron oxidation genes during growth on pyrite. Further investigation into metabolic regulation in *Acidiplasma* may elucidate the reasons for this downregulation and help mitigate its negative impact on pyrite biooxidation. Therefore, the data obtained in this study can be used as a basis for further modifications of extremophilic archaea used in biohydrometallurgy. This, in turn, could lead to improvements in existing biohydrometallurgical technologies.

## 4. Materials and Methods

### 4.1. Strain Growth and Biooxidation Experiment

For transcriptome analysis, we used the acidophilic iron-oxidizing heterotrophic strain *Acidiplasma* sp. YE-1, isolated from the pulp of a bioleaching bioreactor [37]. To prepare biomass samples for RNA extraction and their subsequent analysis, the strain was grown in a liquid mineral medium containing minerals (pyrite, arsenopyrite). The liquid medium, containing the following mineral salts (g/L), was used for growth: (NH_4_)_2_SO_4_—3.0, KCl—0.2, MgSO_4_ × 7H_2_O—0.5, K_2_HPO_4_—0.5. To adjust the initial pH to 1.5, 1.5 mL of concentrated sulfuric acid was added to the medium. Additionally, 0.02% (*w*/*v*) yeast extract was added. Growth was carried out in flasks containing 100 mL of the nutrient medium and 0.5 g of the respective mineral on a rotary shaker (200 rpm) at 50 °C for 10 days. In all experiments, the strain was inoculated to achieve an initial cell number of approximately 1 × 10^7^ cells/mL. Biomass of the strain grown in the same medium without minerals, but supplemented with FeSO_4_ × 7H_2_O—25 g/L, as well as from the same medium amended with 0.5 g of elemental sulfur per 100 mL as substrate, were used as reference samples.

During the growth of the strain, ferrous and ferric iron ion concentrations were measured by trilonometric titration [79]. pH values were determined using pH-150 MI pH meter (Izmeritelnaya tekhnika, Moscow, Russia). Quantitative assessment of microorganisms was carried out by direct counts using an Amplival (Carl Zeiss, Jena, Germany) microscope equipped with a phase contrast device.

Experiments were performed in triplicate.

### 4.2. RNA Isolation

The biomass was collected at the exponential growth stage, as determined by cell count in the liquid medium (on the medium with divalent iron, this corresponded to 1 day of growth, in other cases to 4 days). To isolate RNA, biomass samples were collected by centrifugation (10,000 rpm, 15 min).

Total RNA was isolated using the RNA PowerSoil Total RNA Isolation Kit (MO BIO Laboratories, Inc., Carlsbad, CA, USA). Ribosomal RNA removal was performed using the QIAseq FastSelect-5S/16S/23S Kit (Qiagen, Hilden, Germany). Total RNA concentration was determined using the fluorescence method using the Qubit RNA HS Assay Kit (ThermoFisher, Waltham, MA, USA). The quality of the isolated RNA was assessed via microcapillary electrophoresis using RNA 6000 Pico Kit (Agilent, Santa Clara, CA, USA). cDNA libraries were prepared using NEBNext^®^ mRNA Library Prep Reagent for Illumina (New England Biolabs, Inc., Ipswich, MA, USA) according to the manufacturer’s protocols. cDNA sequencing was performed on an Illumina HiSeq-2500 platform (Illumina, San Diego, CA, USA).

### 4.3. Bioinformatic Analysis

The Cutadapt v4.8 [80] tool was used to remove adapter sequences from Illumina reads, and low-quality read ends were trimmed with Sickle v1.33 [81] (quality threshold q = 30). Reads were mapped to the reference genome (GeneBank: CP133599) using Bowtie2 v2.3.5.1 [82] with the --no-discordant parameter. Gene expression levels were calculated via HTSeq-count v2.0.7 [83] using the “intersection_nonempty” overlap model. Data normalization and statistical testing for differential expression (DE) analysis were performed using the standard workflow of DESeq2 v1.38.3 R package [84]. Genes with an adjusted *p*-value < 0.05 and absolute log2 fold change > 1 were considered DE between conditions. Gene function annotation against the KEGG database was conducted with KofamScan v1.3.0 [85], and the NCBI Conserved Domains Database (CDD) was used to assign genes to COG categories via the rpsblast v2.9.0 tool. The ClusterProfiler v4.6.2 [86] package was used to identify enriched KEGG and COG categories; terms exhibiting an adjusted *p*-value ≤ 0.05 were statistically significant.

### 4.4. Statistical Analysis

Three biological replicates were performed per condition. Data are reported as means ± standard deviations, and MS Excel was employed for statistical analysis. A *p*-value threshold of 0.05 indicated significance.

### 4.5. Nucleotide Sequence Accession Numbers

This BioProject has been deposited in GenBank under accession number PRJNA1229848. The sequences obtained in this project have been deposited in the NCBI Sequence Read Archive under the accession numbers SRX27892493–SRX27892504.

## 5. Conclusions

In the present work, differential gene expression was studied in a thermoacidophilic archaeal strain of the genus *Acidiplasma* using transcriptomic analysis during growth on ferrous iron, elemental sulfur, pyrite, or arsenopyrite. We identified metabolic pathways involved in ferrous iron and sulfur oxidation—which play a key role in sulfide mineral oxidation—and determined how their expression depends on the substrate provided. The blue copper protein sulfocyanin may play an important role in both iron and sulfur oxidation. Furthermore, sulfur oxidation involves genes encoding well-known proteins that participate in the oxidation of reduced inorganic sulfur compounds (RISCs), such as sulfur oxygenase reductase (SOR) and thiosulfate:quinone oxidoreductase (TQO). We also detected the expression of certain genes from the 3-hydroxypropionate/4-hydroxybutyrate (3HP/4HB) carbon dioxide fixation pathway. However, the genome lacks the complete set of genes for the 3HP/4HB pathway, which is consistent with the inability of *Acidiplasma* YE-1 to grow autotrophically. It was found that the presence of pyrite leads to a decrease in the expression level of sulfocyanin, which is accompanied by inhibition of iron oxidation, resulting in a low rate of pyrite bioleaching. This finding is crucial for understanding the mechanisms of interaction between acidophilic microorganisms and sulfide minerals, which may be useful for managing biohydrometallurgical processes.

## Data Availability

All raw data for RNA-seq were deposited into NCBI (BioProject accession code PRJNA1229848).

## Supplementary Materials

The following supporting information can be downloaded at: https://www.mdpi.com/article/10.3390/ijms26199287/s1.
